# SARS-CoV-2 infects blood monocytes to activate NLRP3 and AIM2 inflammasomes, pyroptosis and cytokine release

**DOI:** 10.21203/rs.3.rs-153628/v1

**Published:** 2021-08-11

**Authors:** Caroline Junqueira, Ângela Crespo, Shahin Ranjbar, Mercedes Lewandrowski, Jacob Ingber, Luna B. de Lacerda, Blair Parry, Sagi Ravid, Sarah Clark, Felicia Ho, Setu M. Vora, Valerie Leger, Caroline Beakes, Justin Margolin, Nicole Russell, Kyle Kays, Lee Gehrke, Upasana Das Adhikari, Lauren Henderson, Erin Janssen, Douglas Kwon, Chris Sander, Jonathan Abraham, Michael Filbin, Marcia B. Goldberg, Hao Wu, Gautam Mehta, Steven Bell, Anne E. Goldfeld, Judy Lieberman

**Affiliations:** 1 Program in Cellular and Molecular Medicine, Boston Children’s Hospital, USA; 2 Department of Pediatrics, Harvard Medical School, USA; 3 Instituto René Rachou, Fundação Oswaldo Cruz, Brazil; 4 Department of Medicine, Harvard Medical School, USA; 5 Emergency Medicine, Massachusetts General Hospital Institute for Patient Care, USA; 6 Department of Microbiology, Blavatnik Institute, Harvard Medical School, USA; 7 Department of Biological Chemistry and Molecular Pharmacology, Harvard Medical School, USA; 8 Institute for Medical Engineering and Science, Massachusetts Institute of Technology, USA; 9 Ragon Institute, Massachusetts General Hospital, Massachusetts Institute of Technology, Harvard Medical School, USA; 10 Division of Immunology, Boston Children’s Hospital, USA; 11 cBio Center, Dana-Farber Cancer Institute and Department of Cell Biology, Harvard Medical School, Boston, MA 02215, USA; 12 Center for Bacterial Pathogenesis, Department of Medicine, Division of Infectious Diseases, Massachusetts General Hospital, USA; 13 Institute for Liver and Digestive Health, University College London, UK; 14 Institute of Hepatology, Foundation for Liver Research, London, UK; 15 Department of Clinical Neurosciences, University of Cambridge, UK

**Keywords:** COVID-19, SARS-CoV-2, inflammasome, NLRP3, AIM2, pyroptosis, monocyte, CD16, antibody-dependent phagocytosis

## Abstract

SARS-CoV-2 causes acute respiratory distress that can progress to multiorgan failure and death in a minority of patients. Although severe COVID-19 disease is linked to exuberant inflammation, how SARS-CoV-2 triggers inflammation is not understood. Monocytes and macrophages are sentinel immune cells in the blood and tissue, respectively, that sense invasive infection to form inflammasomes that activate caspase-1 and gasdermin D (GSDMD) pores, leading to inflammatory death (pyroptosis) and processing and release of IL-1 family cytokines, potent inflammatory mediators. Here we show that expression quantitative trait loci (eQTLs) linked to higher *GSDMD* expression increase the risk of severe COVID-19 disease (odds ratio, 1.3, p<0.005). We find that about 10% of blood monocytes in COVID-19 patients are infected with SARS-CoV-2. Monocyte infection depends on viral antibody opsonization and uptake of opsonized virus by the Fc receptor CD16. After uptake, SARS-CoV-2 begins to replicate in monocytes, as evidenced by detection of double-stranded RNA and subgenomic RNA and expression of a fluorescent reporter gene. However, infection is aborted, and infectious virus is not detected in infected monocyte supernatants or patient plasma. Instead, infected cells undergo inflammatory cell death (pyroptosis) mediated by activation of the NLRP3 and AIM2 inflammasomes, caspase-1 and GSDMD. Moreover, tissue-resident macrophages, but not infected epithelial cells, from COVID-19 lung autopsy specimens showed evidence of inflammasome activation. These findings taken together suggest that antibody-mediated SARS-CoV-2 infection of monocytes/macrophages triggers inflammatory cell death that aborts production of infectious virus but causes systemic inflammation that contributes to severe COVID-19 disease pathogenesis.

## One sentence summary

Antibody-mediated SARS-CoV-2 infection of monocytes activates inflammation and cytokine release.

In a small subset of mostly elderly patients and patients with comorbidities, SARS-CoV-2 causes severe COVID-19 disease marked by acute respiratory distress that can progress to multiorgan failure and death ^1^ Severe disease is linked to an overly exuberant inflammatory response, including elevated serum pro-inflammatory cytokines, C-reactive protein, and lactate dehydrogenase (LDH) ^2–6^. Increased chronic inflammation is associated with aging (“inflammaging”) and the comorbidities linked to severe COVID-19 disease ^7^ Myeloid immune cells (monocytes, macrophages, dendritic cells) are sentinels that sound the innate immune alarm by sensing invasive infection and danger to activate inflammasomes ^8^. They are often the most important source of inflammatory cytokines during inflammation, and their activation is required to process and release IL-1 family cytokines, arguably the most potent inflammatory mediators in the body ^9^. However other pathways, including NF-κB activation by Toll-like receptors or the TNF receptor superfamily and T_H_17 lymphocyte cytokines, can also cause severe inflammation. When inflammasomes sense danger or infection, they recruit the ASC adaptor and assemble into large supramolecular complexes that recruit and activate caspase-1, which in turn processes interleukin (IL)-1 family pro-cytokines and the pore-forming protein GSDMD that damages the cell membrane, leading to cell death and inflammatory cytokine release ^8^. Cell membrane rupture during pyroptosis releases cytokines, chemokines and other alarmins that recruit and activate immune cells to sites of infection. Release of large proteins such as the tetramer LDH (144 kDa), is a pathognomonic feature of pyroptosis and other forms of necrotic cell death ^8^. Elevated LDH is one of the best correlates of severe COVID-19 disease ^6^.

## Circulating monocytes and plasma of COVID-19 patients show signs of pyroptosis

**ED Figure 1.**
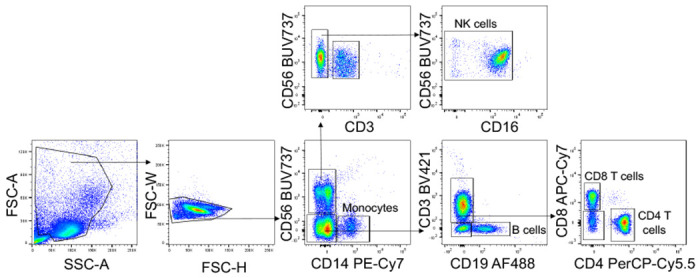
Identification of lymphocyte and monocyte subsets in healthy donors and COVID-19 patients. Gating strategy for identifying lymphocytes and monocytes in Figure 1.

**Figure 1.**
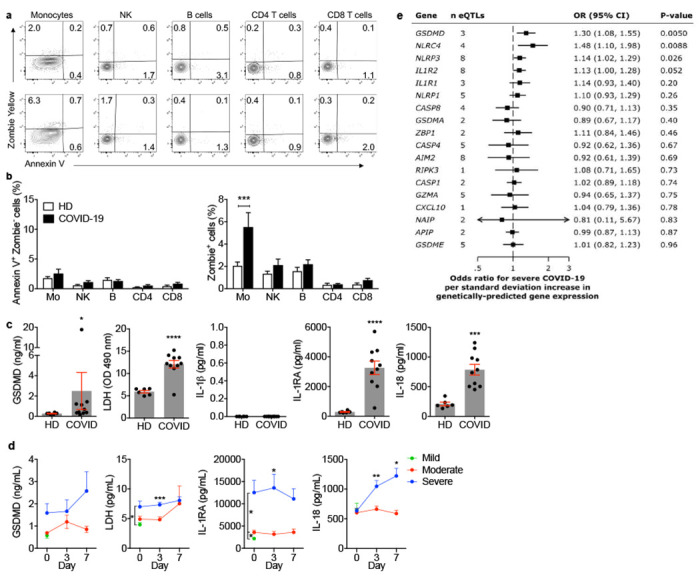
Circulating monocytes from COVID-19 patients are undergoing pyroptosis **a-c**, Representative flow cytometry contour plots (**a**) and percentage of lymphocyte and monocyte subsets staining for Annexin V only or Zombie (**b**) in 19 healthy donors (HD) and 22 SARS-CoV-2 infected patients. **c**, Concentration of gasdermin D (GSDMD), pyroptosis-related cytokines (IL-1β, IL-1RA, IL-18) and lactate dehydrogenase (LDH) activity in the plasma of HD (n=6) or SARS-CoV-2 positive patients (COVID, n=10). **d**, Plasma pyroptosis biomarkers GSDMD, LDH activity, IL-1RA and IL-18 at presentation and during hospitalization in COVID-19 patients with mild, moderate and severe COVID-19 acuity scores (n=60). **e**, Odds ratio for severe COVID-19 disease relative to eQTLs that are associated with increased gene expression of pyroptosis, necroptosis and death receptor-related genes. Data show mean ± S.E.M or odds ratio (95% confidence interval). *p<0.05, **p<0.01, ***p<0.001, ****p<0.0001 by two-way ANOVA (b) Mann-Whitney or Kolmogorov-Smirnov test (c) and multiple t-tests (d).

Because inflammasome activation in myeloid cells is a major mediator of inflammation ^2,10,11^, we examined blood of SARS-CoV-2-infected donors for signs of inflammasome activation and pyroptosis. Freshly isolated mononuclear cells from 19 healthy donors (HD) and 22 COVID-19 patients seen in the emergency department (ED) of Massachusetts General Hospital were stained for hematopoietic cell markers, a small fixable dye (Zombie Yellow) that enters dying cells whose plasma membrane is damaged and annexin V, which identifies cells undergoing programmed cell death (Fig. 1a,b, Extended Data Fig. 1, Table S1). Although annexin V^+^Zombie^−^ apoptotic cells did not increase in any subpopulation in COVID-19 samples, on average ~11% of monocytes of COVID-19 patients took up Zombie dye, a sign of ongoing membrane damage consistent with pyroptosis. Plasma from the same COVID-19 and HD samples was assessed by multiplex ELISA and LDH activity assay for specific inflammatory markers of pyroptosis (GSDMD, IL-1β, IL-1RA, IL-18, LDH) (Fig. 1c) and for other markers of inflammation that are not pyroptosis-specific (inflammatory cytokines IL-6, TNF, IL-17/17A; growth factors IL-7, G-CSF; chemokines CCL7, CXCL9, CXCL10) and for interferons (IFNβ, IFNγ). Consistent with published data ^12–14,15,16^, all the markers of inflammation that are not specific for pyroptosis were significantly elevated in COVID plasma, except for IL-17/17A, and the IFNs were not detected above baseline (data not shown). All of the pyroptosis specific markers, except IL-1β, were significantly elevated in COVID-19 patient plasma compared to HD. Plasma IL-1β was below the level of detection in all samples, which was not surprising since it is rapidly cleared from the blood and usually not detected even in patients with severe disease caused by ongoing pyroptosis, such as those bearing constitutively active *NLRP3* mutations ^9^ or with IL-1-mediated systemic juvenile idiopathic arthritis ^17^ However, its antagonist IL-1RA, which can be used as a surrogate ^9^, was greatly increased in COVID-19 samples. It is worth noting that IL-1 family cytokines and pyroptosis are well known potent activators of the other elevated mediators of inflammation ^18^.

To determine if pyroptosis-related biomarkers correlate with COVID-19 disease severity, plasma from 60 COVID-19 patients who presented to the ED was analyzed for GSDMD, LDH, IL-1RA and IL-18 at presentation and on days 3 and 7 for patients who were hospitalized (Fig. 1d, Table S2). Patients were grouped into mild, moderate or severe disease using the MGH COVID Acuity scale ^19,20^. Moderate disease patients required supplemental O_2_ and severe disease patients required mechanical ventilation or died. Plasma levels of GSDMD, LDH, IL-1RA and IL-18, were all elevated in severe patient samples compared to those with mild or moderate disease, but the increase in GSDMD did not reach significance. Taken together, these results provide evidence of ongoing pyroptosis in blood monocytes and plasma of COVID-19 patients that was more prominent in patients who developed more severe disease.

## *GSDMD* eQTLs increase the risk of severe COVID-19 infection

**ED Figure 2.**
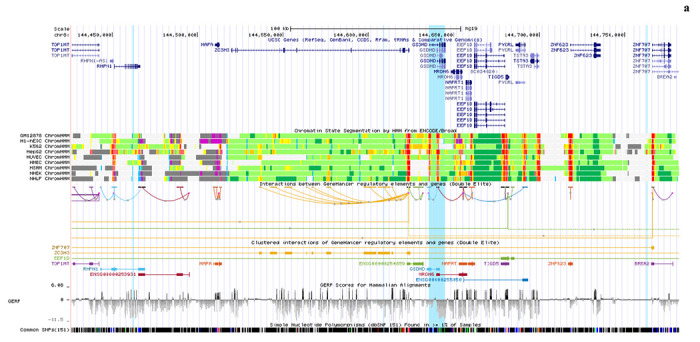

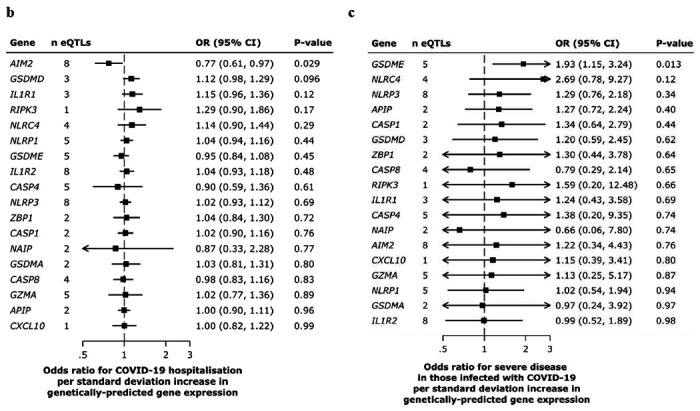
Analysis of genetic link of immune gene eQTLs to severe COVID-19 disease **a,** Screenshot of UCSC genome browser in the vicinity of *GSDMD* (highlighted in turquoise) on chromosome 8. The eQTLs are the thin turquoise vertical lines. None are within the *GSDMD* gene - one is within the gene *RHPN1*, one is within *ZC3H3*, and one is adjacent to *ZNF707.* All are eQTLs (validated by the eQTLGen consortium)^21^ that are associated with increased *GSDMD* expression. **b,** Analysis of eQTL links to COVID-19 hospitalization (6406 hospitalized cases versus 902,088 population controls), **c,** Analysis of eQTL links in patients with severe COVID-19 disease (269 cases) vs control infected patients, who did not require hospitalization for COVID-19 (688 controls).

To probe further whether pyroptosis might be associated with severe COVID-19 infection, we used Mendelian randomization analyses to examine whether expression quantitative trait loci (eQTLs) linked in eQTLGen ^21^ to increased blood expression of 18 immune genes are associated with severe COVID-19 disease. The gene products of the genes analyzed mostly play roles in inflammation and/or programmed necrosis - 11 inflammasome and pyroptosis-related genes: *AIM2, NLRC4, NLRP1, NLRP3, NAIP, CASP1, CASP4, GSDMA, GSDMD, GSDME, GZMA;* 3 genes related to inflammatory cytokine/chemokine signaling: *IL1R1, IL1R2, CXCL10;* 2 necroptosis (another inflammatory programmed necrosis pathway) genes: *RIPK3, ZBP1;* and 2 death receptor or apoptosis signaling genes: *CASP8, APIP.* Data were from case-control study cohorts of the COVID-19 Host Genetics Initiative release #4 (October, 2020) ^22^ (Fig. 1e, Table S3). In the analysis, the contribution of each eQTL for a given gene was weighted according to its prevalence in the general population. Comparing 4336 severe COVID-19 cases with 623,902 population controls, *GSDMD* eQTLs (of which there are 3 (Extended Data Fig. 2a) were most significantly associated with increased COVID-19 respiratory failure (odds ratio 1.30, 95% confidence interval (1.08, 1.55), p<0.005). eQTLs associated with higher expression of two inflammasome genes (*NLRC4* and *NLRP3*) were also significantly linked to severe COVID-19 disease. The odds ratio for the 4 *NLRC4* eQTLs was 1.48 (95% confidence interval (1.10, 1.98), p<0.008) and for the 8 *NLRP3* eQTLs was 1.14 (95% confidence interval (1.02, 1.29), p<0.03). A parallel analysis of eQTL links to COVID-19 hospitalization (6406 hospitalized cases versus 902,088 population controls, Extended Data Fig. 2b and Table S3) identified only 1 significant association with *AIM2*, another inflammasome gene, which has 8 eQTLs. However, in this case *AIM2* eQTLs associated with increased COVID-19 hospitalization led to a lower risk of COVID-19 hospitalization (odds ratio 0.77, 95% confidence interval (0.61,0.97), p<0.03). When a smaller dataset compared COVID-19 patients with severe vs mild disease (269 cases of severe COVID-19 with 688 controls of non-hospitalized COVID-19 patients) (Extended Data Fig. 2c and Table S3), patients with severe COVID-19 disease had significantly more eQTLs linked to higher expression of only one gene, the gasdermin *GSDME* with 5 eQTLs (odds ratio 1.93, 95% confidence interval (1.15, 1.32), p<0.013). In all of these analyses, eQTLs for the 7 analyzed genes that were not directly associated with inflammasome activation and pyroptosis were not significantly enriched in patients with more severe COVID disease. Thus, the genetic link between increased gasdermin and inflammasome eQTLs and severe COVID-19 infection further supports a role for pyroptosis in clinical deterioration. The stronger genetic link between GSDMs and inflammasomes in severe COVID disease patients compared to less severe COVID-19 hospitalized patients suggested by our analysis (despite the larger sample size for the hospitalized patient analysis), hints that pyroptosis may be especially important in the immunopathogenesis that accompanies the transition from initial pneumonitis to respiratory failure and systemic disease.

## Circulating monocytes have activated NLRP3 and AIM2 inflammasomes

**ED Figure 3.**
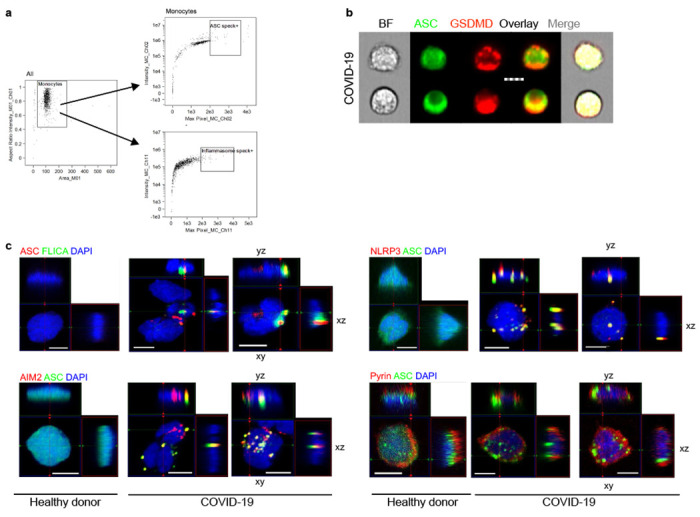
**a,** Gating strategy for imaging flow cytometry analysis of isolated monocytes. **b,** Representative imaging flow cytometry images of GSDMD and ASC staining in COVID-19 patient monocytes that lacked ASC specks. Scale bar, 7 μm. **c,** Representative confocal image z-stacks and plane projections of monocytes of HD and COVID-19 patient monocytes, stained for the same markers as in Figure 2. Scale bars, 5 μm.

**Figure 2.**
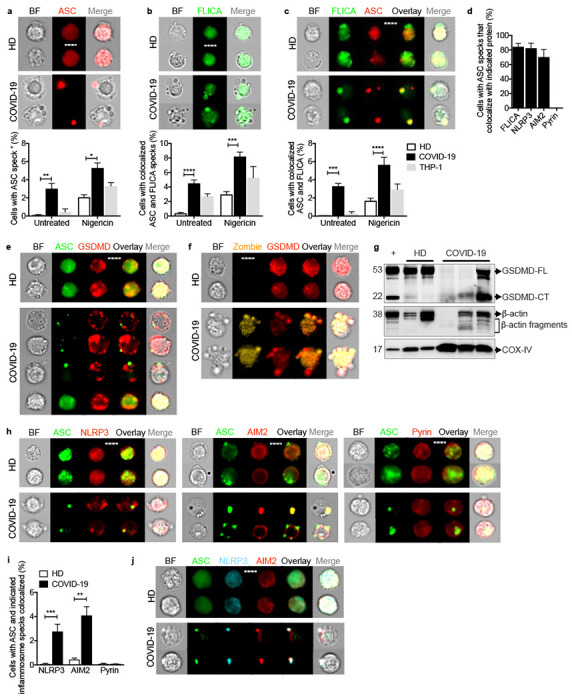
Monocytes from COVID-19 patients have activated inflammasomes, caspase-1 and gasdermin D Circulating monocytes from healthy donors (HD) or COVID-19 patients were treated or not with nigericin, and then analyzed by imaging flow cytometry for caspase-1 activation (by FLICA assay before fixation) and fixed and stained for the indicated markers, **a-c,** Percentage of monocytes with activated ASC, n=5 (**a**) or caspase-1, n=5 (**b**) or colocalized ASC/caspase-1 specks, n=5 (**c**). Representative images are shown at top and quantification is graphed at bottom. **d,** Percentage of ASC-speck-containing monocytes with colocalized activated caspase-1 (n=6), NLRP3 (n=6), AIM2 (n=4), or pyrin (n=4) specks. **e,f,** Representative images of ASC (**e**) and Zombie dye (**f**) and GSDMD co-stained monocytes (4 independent experiments). **g**, Immunoblot of lysates of freshly isolated purified HD and COVID-19 monocytes and of HD monocytes treated with LPS and nigericin (+) probed with GSDMD mAb that recognizes full length (GSDMD-FL) and the cleaved C-terminal fragment (GSDMD-CT) (top). Anti-β-actin (middle) and COX-IV (bottom) are loading controls. **h,i** Representative images of ASC co-staining with NLRP3 (left), AIM2 (middle) and pyrin (right) (**h**) and quantification of monocytes showing colocalization of ASC specks with each inflammasome (**i**). **j**, Representative images of co-staining of ASC, NLRP3, and AIM2 (from 3 independent experiments). Scale bar, 7 μm. BF, brightfield. Mean ± S.E.M. is shown. *p<0.05, **p<0.01, ***p<0.001, ****p<0.0001 by two-way ANOVA with Tukey’s multiple comparisons test (a-c,i) and by one-way ANOVA with Tukey’s multiple comparisons test (d).

These data suggested that circulating monocytes in COVID-19 patients might die of pyroptosis and release inflammatory cytokines to cause cytokine storm and contribute to poor outcome. Not much is known about how viruses interact with the 27 potential human canonical inflammasome sensors (22 NOD-like receptors (NLR), 4 AIM2-like receptors (ALR) and pyrin) ^8^. The NLRP3 inflammasome, which detects K^+^ efflux generated by a variety of stimuli including extracellular ATP, bacterial toxins or disruption of the cell membrane, could be activated by lytic SARS-CoV-2 infection itself or by specific viral proteins ^23,24^ Three SARS-CoV-2 proteins, Orf3a, Orf8b and the E envelope, are “viroporins” (ion channels) that potentially activate NLRP3 by K^+^ efflux when ectopically expressed ^25–28^. Orf3 and Orf8 are encoded by the pathogenic, but not the avirulent, human CoVs. Interestingly, bats, the natural zoonotic hosts of SARS-CoV and SARS-CoV-2, have a dampened NLRP3 response to infection with multiple viruses, including MERS-CoV, which might explain their ability to tolerate these infections despite high viral loads ^29,30^. To probe whether circulating monocytes from COVID-19 patients are undergoing pyroptosis, freshly isolated, enriched monocytes from 3-8 HD or COVID-19 patients with mixed disease severity (Extended Data Fig. 3a) were analyzed by imaging flow cytometry for expression and intracellular distribution of the common inflammasome adapter ASC, activated caspase-1 (by fluorochrome-labeled inhibitor of caspases assay (FLICA)) and GSDMD. Canonical inflammasome activation forms a large micron-sized inflammasome-ASC-caspase-1 speck ^8^. About 3% of fresh monocytes from COVID-19 patients, but none in HD, had activated caspase-1 and ASC specks (Fig. 2a–c). Most of the cells with ASC specks (>80%) also had co-localized activated caspase-1 (Fig. 2d). When these monocytes were treated with a low concentration of nigericin (20 μΜ) for 30 minutes, which triggers the NLRP3 inflammasome, all the samples had some cells with ASC-caspase-1 specks, but the COVID-19 patient samples had about three times more, indicating that they were more prone to stimulated pyroptosis. As a control, a monocytic cancer cell line (THP-1) was treated with nigericin.

Many of the fresh monocytes with ASC specks from COVID-19 samples showed ballooning plasma membranes and GSDMD redistribution from the cytoplasm to form prominent cell membrane puncta, consistent with GSDMD pore formation and pyroptosis, but cells without ASC specks did not (Fig. 2e,f, Extended Data Fig. 3b). Immunoblots of lysates of freshly isolated HD and COVID-19 patient monocytes and of LPS plus nigericin-treated HD monocytes were probed for full-length GSDMD (GSDMD-FL) and its cleaved C-terminal fragment (GSDMD-CT) and the housekeeping proteins, β-actin and COX-IV (Fig. 2g). During pyroptosis, cleaved GSDMD and actin are released, the actin cytoskeleton disintegrates and pyroptotic cells no longer stain for actin, while membrane-bound proteins, like COX-IV, are mostly retained ^31,32^. Although GSDMD-FL was observed in all HD samples, it was only detected in 1 of 3 COVID-19 samples. A prominent GSDMD-CT fragment was detected in COVID-19 monocytes and in the positive control, LPS + nigericin-treated HD monocytes. Although mitochondrial inner membrane-anchored COX-IV was detected in all the samples, FL β-actin was not detected in one of the COVID-19 samples, but immunoreactive β-actin fragments were detected in all the COVID-19 samples and in nigericin-activated HD monocytes. Thus, circulating monocytes from COVID-19 patients show signs of GSDMD cleavage and pyroptosis.

To identify the activated inflammasome sensor, fresh HD and COVID-19 monocytes were co-stained for ASC and 3 canonical inflammasomes (NLRP3, AIM2 (activated by cytoplasmic DNA) or pyrin (activated by bacterial toxins) (Fig. 2d, h–j) ^33,34^ In COVID-19 patient monocytes ASC specks co-localized with NLRP3 and AIM2, but there were no pyrin specks. The activation of AIM2 was unexpected, although AIM2 has been shown, in rare cases, to be activated by RNA viruses by an unclear mechanism ^35–37^ AIM2 might sense host genomic or mitochondrial DNA since mitochondrial membranes are damaged during pyroptosis ^38^. Almost all of the cells with ASC specks had co-localized NLRP3 and AIM2 specks (Fig. 2d) and ASC, NLRP3 and AIM2 colocalized (Fig. 2j). We did not expect to find more than one inflammasome sensor stimulated in the same cell, although co-localization of 2 distinct inflammasome sensors to the same speck has been previously observed ^39^. Confocal microscopy confirmed ASC, activated caspase-1, NLRP3 and AIM2 colocalization in inflammasomes selectively in COVID-19 monocytes (Extended Data Fig. 3c). These data showing NLRP3- and AIM2-ASC-caspase-1 inflammasomes and GSDMD membrane localization and cleavage, together with our detection of dying Annexin V^−^Zombie^+^ circulating monocytes and plasma GSDMD and IL-1 family cytokines (Fig. 1), indicate that some COVID-19 blood monocytes are dying of pyroptosis.

## Circulating monocytes are infected with SARS-CoV-2 and infected cells are undergoing pyroptosis

**ED Figure 4.**
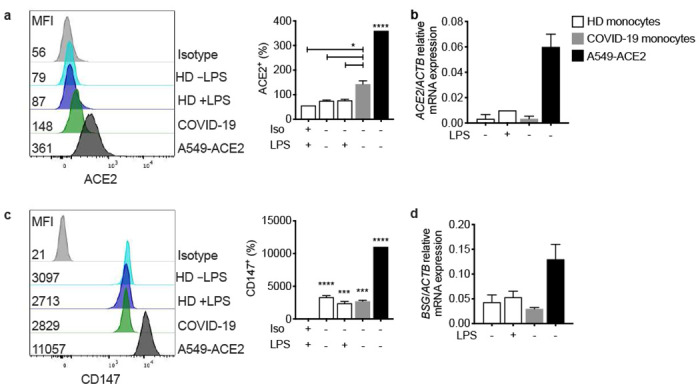
ACE2 and CD147 expression on circulating monocytes. Purified blood monocytes from HD (n=3) and COVID-19 patients (n=4) were analyzed by flow cytometry (**a,c**) or qRT-PCR (**b,d**) for expression of ACE2 (**a,b**) or CD147 (*BSG*) (**c,d**). HD monocytes were treated or not with LPS before analysis. A549-ACE2 cells were used as positive control. Mean ± S.E.M. is shown. *p<0.05, **p<0.01, ***p<0.001, ****p<0.0001 relative to isotype control-stained LPS activated HD monocytes (a,c) by one-way ANOVA with Tukey’s multiple comparisons test.

**ED Figure 5.**
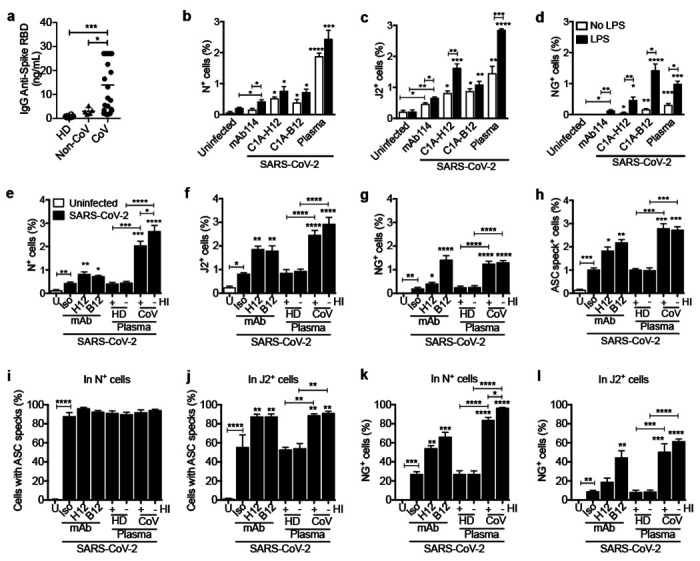
Effect of anti-Spike monoclonal antibodies or pooled COVID plasma and LPS activation on in vitro infection of healthy donor purified monocytes with icSARS-CoV-2-mNG **a,** Spike RBD-specific IgG were quantified by ELISA from the plasma of healthy donor (HD=10), non-COVID-19 patients (n=5) and COVID-19 patient plasma (n=18). **b-l,** HD monocytes were primed (black bars) or not (white bars) with LPS, infected with icSARS-CoV-2-mNG (MOI, 1), then stained 48 h later for nucleocapsid (N) or dsRNA (J2) and ASC and analyzed by imaging flow cytometry. Before infection, virus was preincubated with indicated monoclonal antibodies (IgG1 isotype control mAb114 (Iso)), non-neutralizing anti-spike (C1A-H12 (H12)) or neutralizing anti-RBD (C1A-B12 (B12)) or with pooled HD or COVID-19 patient plasma that had been heat-inactivated (HI) or not. U, uninfected. Quantification of HD monocyte staining for N (**b,e**), J2 (**c,f**), NG (**d,g**) or ASC specks (**e**). **f,g**, Percentage of N^+^ (**i**) and J2^+^ (**j**) cells that had ASC specks. **k,l**, Percentage of N^+^ (**h**) and J2^+^ (**i**) cells that had detectable NG. Mean ± S.E.M. is shown. *p<0.05, **p<0.01, ***p<0.001, ****p<0.0001 by one-way ANOVA with Tukey’s multiple comparisons test (a), relative to Iso or as indicated, by two-way ANOVA with Sidak’s multiple comparisons test (b-d) and by one-way ANOVA with Tukey’s multiple comparisons test (e-l).

**Figure 3.**
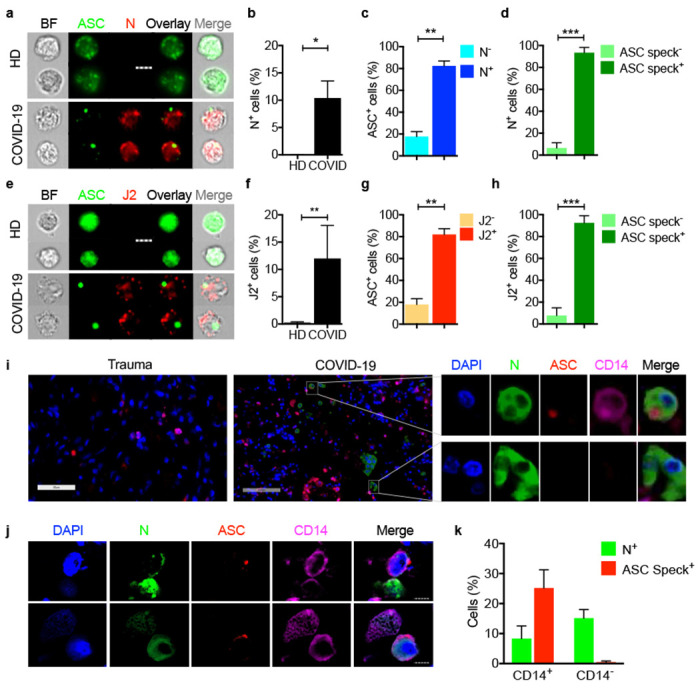
SARS-CoV-2 infects circulating monocytes and lung macrophages and infection activates inflammasome formation **a-h,** Circulating monocytes from HD and COVID-19 patients were purified and stained for SARS-CoV-2 nucleocapsid (N) (n=5) (**a-d**) or dsRNA (J2 antibody) (n=4) (**e-h**) and ASC. Shown are representative imaging flow cytometry images (**a,e**), quantification of percentage of cells that were infected by N (**b**) or J2 (**f**) staining, percentage of uninfected or infected cells (by J2 or N staining) that showed ASC specks (**c, g**) or percentage of cells with or without ASC specks that showed N (**d**) or J2 (**h**) staining, **i-k,** Lung autopsy samples from 4 COVID-19 patients and a control trauma victim without lung disease were stained for N (green), ASC (red), CD 14 (magenta) and DAPI (blue), **i,** Digital scanner images of trauma patient (left) and a representative COVID-19 (middle) lung sample showing magnified image of representative infected CD14^+^ (top) and CD14^−^ (bottom) cells from the COVID-19 sample (right). **j,** Representative confocal microscopy images of infected CD14^+^ cells from COVID-19 patients. **k,** Quantification of N and ASC specks in CD14^+^ and CD14^−^ cells from COVID-19 lungs (n=4). Scale bar, 7 μm (a,e,j). BF, brightfield. Mean ± S.E.M. is shown. *p<0.05, **p<0.01, ***p<0.001 by nonparametric unpaired *t*-test (Mann-Whitney or Kolmogorov-Smirnov).

But what activates inflammasomes in COVID-19 patient monocytes? Since inflammasomes sense invasive infection, the monocytes might be infected. A few recent reports suggest that monocytes ^14,40^ and tissue macrophages ^41^ can be infected with SARS-CoV-2. However, monocytes are generally thought not to express ACE-2, the viral receptor for entry ^42,43^. Indeed, ACE-2 was not detected or barely detected by flow cytometry and qRT-PCR on both healthy donor (HD) monocytes, even when they were activated, and COVID-19 monocytes (Extended Data Fig. 4a,b). Both HD and COVID-19 patient monocytes also expressed similar levels of CD147 (basigin or EMMPRIN), an immunoglobulin superfamily receptor implicated in bacterial, parasite and viral entry, which has been reported to bind to SARS-CoV-2 spike protein and facilitate viral uptake and infection ^44^ (Extended Data Fig. 4c,d). Monocytes express 2 Fcγ receptors – CD32 (FcγRII, expressed on most blood monocytes ^45^) and CD16 (FcγRIII, expressed on a small minority of blood monocytes that are activated and increased in number in COVID-19 patients ^13^. These receptors could recognize antibody-opsonized viral particles and mediate their entry via antibody-dependent phagocytosis (ADP) ^46^. Anti-SARS-CoV-2 spike protein antibodies are detected early in SARS-CoV-2 infection, about the same time as patients start developing inflammatory symptoms ^41,47–49^, and anti-Spike RBD antibodies were detected by ELISA in the plasma of most of the 18 COVID-19 patients we assayed when they presented to the hospital ED (Extended Data Fig. 5a). To examine whether COVID-19 patient blood monocytes are infected, we co-stained isolated HD and COVID-19 patient monocytes for SARS-CoV-2 nucleocapsid (N) (Fig 3a–d) or dsRNA (J2 antibody) (Fig 3e–h) and ASC. Cells that passively take up virions or contain replicating virus could stain for N, but J2 staining indicates active infection ^50^. HD monocytes did not stain for N, dsRNA or ASC. About 10% of COVID-19 patient blood monocytes stained for N or dsRNA (Fig 3b,f), indicating that circulating monocytes in COVID-19 patients are infected. Moreover, virtually all the infected cells showed ASC specks (Fig 3c,g) and virtually all the ASC speck^+^ cells were infected (Fig 3d,h). Thus SARS-CoV-2 infection of monocytes activates inflammasomes and pyroptosis.

## Lung macrophages are infected in COVID-19 autopsies and have activated inflammasomes

Since the lung and airways are the main site of infection, we next assessed whether cells in lung autopsy specimens that stain for CD14, a marker of tissue macrophages and dendritic cells, were infected with SARS-CoV-2 and had active inflammasomes. Fixed slides from four human autopsy specimens and one uninfected trauma victim without lung pathology were co-stained for CD14, ASC, SARS-CoV-2 N and DAPI (Fig 3i–k). In the COVID-19 lungs, 8.3±4.2 CD14^+^ cells and 15.1±2.9 CD14^−^ cells stained for N and were infected, but no infection was detected in the control trauma victim lung. As expected, N staining was detected in both E-cadherin^+^ epithelial and CD31^+^ endothelial CD14^−^ cells selectively in the infected lungs (data not shown). However, ASC specks were detected only in CD14^+^, but not in CD14^−^, COVID-19 lung cells, suggesting that tissue-resident macrophages have activated inflammasomes, but infected lung epithelial and endothelial cells do not. We also did not detect ASC specks in the uninfected control autopsy specimen. About a quarter of the CD14^+^ lung cells had ASC specks, although only ~8% of them were N^+^. This discrepancy suggests that inflammasomes in uninfected macrophages in infected lungs may be activated by danger-associated molecular patterns (DAMPs), such as cellular alarmins like HMGB1 or ATP, released from infected and/or other damaged cells in the tissue. Identifying the noninfectious stimulators of inflammasome activation of macrophages in the lung will require further study.

## CD16 uptake of antibody-opsonized virus infects healthy donor monocytes

**Figure 4.**
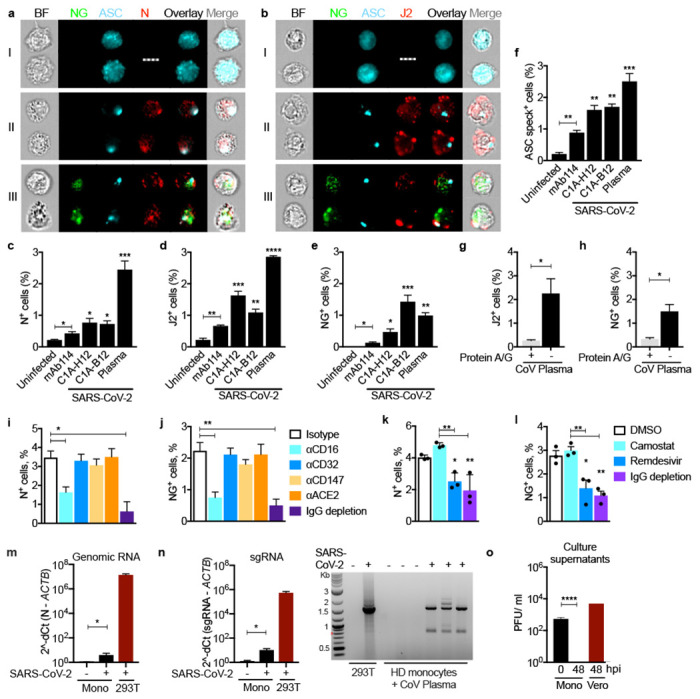
Healthy donor monocytes take up antibody-opsonized SARS-CoV2 via the Fc receptor CD16, begin viral replication but do not produce infectious virus Healthy donor (HD, n=3) monocytes were primed with LPS, infected with icSARS-CoV-2-mNG and stained 48 h later for nucleocapsid (N) or dsRNA (J2) and ASC. Before infection, virus was preincubated with IgG1 isotype control mAb114, non-neutralizing anti-spike (C1A-H12) or neutralizing anti-RBD (C1A-B12) or with pooled COVID-19 patient plasma. Antibodies or plasma were present throughout the culture. **a,b**, Representative imaging flow cytometry images of (I) uninfected monocytes, (II) N or J2 staining cells without detectable Neon green (NG), or (III) with NG and N or J2 staining. **c-f,** Quantification of HD monocyte staining for N (**c**), J2 (**d**) NG (**e**) or ASC specks (**f**). **g,h**, Percentage of J2+ (**g**) and NG+ (**h**) cells in LPS-activated HD monocytes after infection with icSARS-CoV-2-mNG virus that was preincubated with COVID-19 pooled plasma that was depleted or not of immunoglobulins using Protein A/G beads. **i-l,** SARS-CoV-2, preincubated with pooled COVID-19 plasma that was depleted or not of immunoglobulins, was used to infect LPS-treated HD monocytes in the presence of indicated blocking antibodies (**i,j**) or antiviral drugs (**k,l**) and infection was assessed 48 h later by flow cytometry detection of N **(i,k)** or NG **(j,l)**. **m,n,** qRT-PCR analysis of genomic SARS-CoV-2 N RNA **(m)** and subgenomic (sg)RNA **(n, left)** in uninfected or infected HD monocytes, normalized to *ACTB* mRNA. Infected HEK293T were used as positive control. Agarose gel electrophoresis of ethidium bromide-stained qRT-PCR subgenomic products is shown **(n, right)**. The ~1600 nt band present only in the COVID-19 samples was excised and sequenced and confirmed to represent the subgenomic RNA for the *N* gene. **o,** SARS-CoV-2 plaque forming units (PFU) in the culture supernatants of infected monocytes at time 0 and 48 h post infection (hpi) and of infected Vero E6 cells. BF, Brightfield. Scale bar, 7 μm. Mean ± S.E.M. is shown. *p<0.05, **p<0.01, ***p<0.001, ****p<0.0001 by two-way ANOVA with Sidak’s multiple comparisons test (c-f), nonparametric unpaired *t*-test (g,h) and one-way ANOVA (i-o).

To confirm that monocytes can be infected by SARS-CoV-2, purified HD monocytes were infected with an engineered infectious clone (icSARS-CoV-2-mNG) derived from the 2019-nCoV/USA_WA1/2020 strain encoding a Neon Green (NG) fluorescent protein gene as a reporter of viral replication ^51^. Monocytes, primed or not with LPS, were infected (MOI 1) with reporter virus that was preincubated with IgG_1_ isotype control antibody (mAb114), anti-Spike mAbs (non-neutralizing C1A-H12, neutralizing C1A-B12) ^52^ or pooled HD or COVID-19 patient plasma (heat inactivated or not). Antibodies and plasma were also present during culture. After 48 h, monocytes were co-stained for dsRNA or SARS-CoV-2 N and ASC and analyzed by imaging flow cytometry (Fig. 4a–f, Extended Data Fig. 5b–h). Staining for N indicates virus internalization, whereas J2 staining and NG fluorescence indicate virus replication. Without LPS or anti-Spike antibody or COVID-19 pooled plasma, few HD monocytes took up or replicated the virus, but infection increased significantly in the presence of anti-Spike mAb or COVID-19 plasma. Nonetheless, N, J2 and NG positive monocytes were reproducibly detected at low levels above background after HD monocyte infection with virus preincubated with isotype control mAb or with HD plasma, suggesting that there is inefficient anti-SARS-Cov-2 antibody-independent uptake and infection of monocytes in addition to more efficient infection with spike-antibody opsonized virus. The highest in vitro infection rate was ~3% in HD monocytes pretreated with LPS and incubated with patient plasma. N and J2 staining were comparable across the different conditions with a low background of ~0.1% in uninfected samples; fewer cells were NG fluorescent (about half as many) and there was no background NG fluorescence in uninfected samples. More of the J2 or N staining cells in the samples with the highest infection rates (treated with LPS and patient plasma or anti-Spike antibodies) were also NG fluorescent. NG may be less often detected than N or dsRNA because it is highly expressed later in the viral lifecycle and/or is more difficult to detect than N or double-stranded RNA. Nonetheless detection of NG is another indication of active viral replication in monocytes. ASC specks were barely detected in uninfected control HD monocytes but increased with SARS-CoV-2 infection (Fig. 4f, Extended Data Fig. 5i–l). More cells were ASC speck^+^ when SARS-CoV-2 was preincubated with neutralizing or non-neutralizing anti-Spike than with isotype control antibody and still more when virus was preincubated with pooled patient plasma.

The neutralizing activity of the antibody did not consistently affect infection. Heat inactivation of COVID-19 plasma did not reduce infection (Extended Data Fig. 5e–h), suggesting that complement did not facilitate infection. HD plasma or isotype control antibody only weakly increased infection (Extended Data Fig. 5e–h), suggesting that opsonization of virus with anti-viral antibodies might be required for efficient infection. Indeed, immunoglobulin (Ig)-depletion of COVID-19 plasma nearly abrogated viral infection assessed by J2 staining and NG fluorescence (Fig. 4g,h). To identify the monocyte receptor responsible for viral uptake, purified HD monocytes were infected with the reporter virus in the presence of COVID-19 patient plasma that was depleted or not of IgG or in the presence of blocking antibodies to potential monocyte receptors - ACE-2, CD147 and the two FcRs, CD16 and CD32 (Fig. 4i,j). Either blocking CD16 or Ig depletion strongly inhibited infection, while blocking the other receptors had no significant effect. Thus, SARS-CoV-2 infection of monocytes is mostly mediated by CD16 uptake of opsonized virus.

## SARS-CoV-2 infection of monocytes is aborted

Detection of dsRNA and the fluorescent NG reporter strongly suggested that SARS-CoV-2 is not just taken up by monocytes, but also begins to replicate. To confirm viral replication in monocytes and verify that viral uptake is not mediated by the canonical ACE-2 receptor, HD monocytes were infected in the presence of COVID-19 plasma and the antiviral drugs, Remdesivir, an inhibitor of the viral RNA-dependent RNA polymerase, and Camostat mesylate, an inhibitor of the TMPRSS2 serine protease, which primes the Spike protein for ACE-2 mediated entry ^53^ (Fig. 4k,l). Infection, assessed by counting N or NG positive cells, was not affected by Camostat, but was significantly and comparably inhibited by Ig depletion or Remdesivir, confirming antibody-dependent, ACE-2-independent uptake and viral replication. Early in viral replication, a series of plus strand subgenomic RNAs are transcribed with a common leader sequence that are highly specific indicators of viral replication ^30^. To further confirm viral replication, qRT-PCR was used to detect genomic and subgenomic SARS-CoV-2 RNAs. Genomic and subgenomic RNA were assessed after qRT-PCR amplification in SARS-CoV-2-infected HD monocytes using primers to the N1 region of the N gene and to the shared leader sequence and 3’UTR sequences of the subgenomic RNAs, respectively. Genomic and subgenomic RNAs were detected in SARS-CoV-2-infected HD monocytes, but not in uninfected monocytes (Fig. 4m,n). The most abundant amplified sgRNA fragment migrated on agarose gels at the expected size of the N gene subgenomic RNA (1560 nt), and its identity was confirmed by sequencing the excised band. Although multiple assays indicated that monocytes initiate viral replication, we next wanted to know if infected monocytes release infectious virus. Previous studies have not cultured SARS-CoV-2 from COVID-19 plasma, which we confirmed in 9 COVID-19 plasma samples, suggesting that monocyte infection may not produce infectious virus. Indeed, when culture supernatants of infected HD monocytes, harvested just after infection or 48 hours post infection (hpi), we could detect infectious virus that formed plaques in Vero E6 cells in culture supernatants only after adding the virus stock but not 48 hours later (Fig. 4o). Thus, monocyte infection does not produce infectious virus.

## Discussion

Here we show that antibody-opsonized SARS-CoV-2 can infect and replicate in blood monocytes and lung macrophages. As many as 10% of circulating monocytes and 8% of lung macrophages in COVID-19 patients in our study were infected with SARS-CoV-2 and a comparable number of circulating monocytes had activated inflammasomes and took up a small membrane-impermeable dye, indicating that they were undergoing pyroptosis. This is a very large number, considering that dying cells are usually difficult to detect in vivo since they are rapidly eliminated from the body. It may be surprising that monocyte infection and cell death has not been widely recognized. However, we think this is due to three reasons – (1) many studies of COVID-19 blood cells use thawed, frozen cells, and dying or activated cells do not survive freeze-thawing, (2) published studies have not specifically looked at whether circulating mononuclear cells are dying and (3) few researchers have looked at whether monocytes might be infected because they were not thought to express ACE2. In support of our findings, a few studies have shown evidence of increased IL-1 family cytokines in COVID-19 patient plasma, in vitro SARS-CoV-2 entry in myeloid cells or NLRP3 inflammasome-caspase-1 activation in COVID-19 patient blood cells ^12–14,40^. However, none of these studies has shown that SARS-CoV-2 infection of monocytes is antibody-mediated, identified the receptor responsible for viral uptake or shown evidence of viral replication without production of infectious virions. Moreover, no previous study identified SARS-CoV-2 infection of monocytes as the cause of inflammasome activation or showed evidence of ongoing pyroptosis.

We found a one-to-one correspondence between monocyte infection and inflammasome-caspase-1 activation. Inflammasome activation and pyroptosis are likely responsible for aborting viral infection before infectious viruses are fully assembled because a viable host cell is needed to complete replication. Given the high frequency of infected monocytes, induction of pyroptosis in most patients is a protective response that reduces viral burden. The activation of pyroptosis in infected myeloid cells also sounds a potent immune alarm that recruits and activates innate and adaptive immune cells to sites of infection, including the lung, and contributes to immune defense. However, pyroptotic myeloid cells are likely to be a major cause of the serious inflammatory sequelae that lead to acute lung injury, multiorgan damage, vascular leak and respiratory distress in the minority of patients with severe disease.

Four times as many lung-resident macrophages appeared to have inflammasome activation as were infected (as assessed by staining for viral nucleocapsid). Further studies will be needed to identify what stimulates inflammation in uninfected macrophages. Another intriguing finding of this study was the absence of evidence of inflammasome activation in lung epithelial cells compared to our finding of inflammasome activation in virtually every infected monocyte. Why lung epithelial cells resist inflammasome activation will also require further study. Are the genes needed for inflammasome activation, the inflammasome sensors or ASC not adequately expressed? In fact, the lung epithelial cells in autopsy specimens did not stain appreciably for ASC. Another possibility is that an uncharacterized viral ORF might suppress the inflammasome activation pathway selectively in lung epithelial cells.

SARS-CoV-2-infected monocytes had detectable NLRP3 and AIM-2 inflammasomes that recognize cell membrane damage and cytosolic DNA, respectively. Further work is needed to understand how these inflammasomes get activated by SARS-CoV-2 and whether inflammasome activation is restricted to virulent coronaviruses. It will also be worth studying whether other inflammasomes are activated, such as NLRP1, which was recently shown to sense dsRNA ^54^, or whether other viral infections activate multiple inflammasomes. The significant eQTL link of severe COVID disease to the *NLRC4* gene, which encodes an inflammasome that binds to the NAIP sensor of bacterial Type III secretion ^55^ hint that this inflammasome may play a role in severe COVID-19 disease, but if and how it does will need to be explored.

At the time of diagnosis, plasma biomarkers of pyroptosis, including IL-1RA, IL-18, LDH and GSDMD, correlated with development of severe disease. This finding suggests that they might be incorporated into a diagnostic panel to help predict who might be susceptible to overexuberant inflammatory complications and benefit from immune modulating therapy. Repurposing FDA-approved drugs that inhibit inflammatory cytokines or GSDMD, the final mediator of both cytokine release and inflammatory death, are worth assessing in controlled clinical trials. Although anti-IL-1β (canakinumab) did not meet its endpoint for efficacy in hospitalized hypoxic COVID-19 patients (Novartis press release, 11/06/2020), an unpublished manuscript of a randomized control trial of anakinra (IL-1RA) in patients with SARS-CoV-2 pneumonia showed a highly significant reduction in the development of severe respiratory failure and overall clinical severity ^56^. The possible efficacy of antagonizing IL-1 implicates GSDMD activation in severe COVID-19 disease since IL-1 secretion depends on GSDMD pore formation. Antagonists of IL-6 signaling, however, have had weak, at best, effects on COVID-19 infection ^57,58^. The disappointing results of inhibiting IL-6 may be due to suboptimal timing (it is hard to stop a fulminant inflammatory cascade once it has started) or because IL-6 is only one of many inflammatory mediators that are released and increased during severe disease. Two FDA-approved inhibitors of GSDMD, the critical mediator of pyroptosis and IL-1 family cytokine release - disulfiram (Antabuse) ^59^ or dimethyl fumarate (Tecfidera) ^60^ - are also worth evaluating and are currently being evaluated in clinical studies (NCT04485130, NCT04594343, NCT04381936). It is worth noting that administering disulfiram or dimethyl fumarate in mouse models of sepsis, which has many overlapping features with severe COVID-19 disease, strongly improved not only survival, but also plasma levels of IL-6 and TNF.

In human studies like this, it is difficult to assess how much monocyte and macrophage infection and inflammasome activation contribute to COVID-19 inflammation, cytokine release syndrome and severe disease. However, given the large percentage of infected cells, the large number of monocytes in the blood (~1-3 x10^9^), the fact that a quarter of lung macrophages appear to have activated inflammasomes and that myeloid cells are the major source of IL-1 and other inflammatory cytokines, it is likely that monocyte/macrophage infection and inflammasome activation are important contributors to the pathogenesis of severe COVID-19 disease. The relative importance of blood monocytes versus tissue macrophages in inflammation and its serious consequences is also not clear and will require further study. However, it will also be worthwhile to study other infected cells that express GSDMD as sources of inflammation, and to understand what aspects of monocyte/macrophage activation enhance SARS-CoV-2 infection. The significant link we found of *GSDME* eQTLs to severe COVID-19 infection even in a small case-control study (269 cases, 688 controls) suggests that it will also be worth identifying cell populations, infected or not, that have cleaved and activated GSDME in COVID-19 patients. GSDME-expressing cells switch from noninflammatory apoptosis to inflammatory pyroptosis, when they are exposed to apoptotic stimuli or to granzyme B during cytotoxic lymphocyte attack ^8^. Although there is yet no evidence that SARS-CoV-2 activates apoptotic caspases, cells infected with other virulent coronaviruses (SARS-CoV, MERS-CoV and mouse hepatitis virus (MHV)) have been shown to induce apoptosis ^3^.

Our findings implicate opsonizing antibodies in monocyte SARS-CoV-2 infection and inflammasome activation and raise the possibility that antibodies contribute to deleterious immune reactions associated with severe disease ^61,62^. Antibodies are clearly beneficial for blocking infection of ACE-2-expressing lung and airway epithelia, where the virus completes replication to produce infectious progeny, but their Fc regions also mediate cellular uptake and complement activation. Here we show that FcR-mediated uptake of antibody-coated virus triggers pyroptosis, which is a double-edged sword. Uptake into monocytes/macrophages is a dead end for the virus - it removes virions from the extracellular milieu, blocks them from producing infectious progeny and prevents them from disseminating to cells it can productively infect. On the other hand, the inflammatory mediators spewed out from pyroptotic monocytes and macrophages can cause severe inflammatory side effects and cytokine storm. It may not be a coincidence that clinical deterioration coincides temporally with the detection of SARS-CoV-2 antibody responses ^41,47–49^. Patients with severe COVID-19 disease have recently been shown to have a strong increase in antiviral IgGs that are afucosylated in their Fc region and bind more strongly to CD16, the main receptor that may be responsible for uptake of antibody-opsonized SARS-CoV-2 ^61,63,64^ Characterizing how antibody features, such as glycosylation and choice of constant region, change the ratio of protective vs deleterious functions of anti-spike antibodies will be important not only for understanding SARS-CoV-2 pathogenesis, but also for choosing the best preparations of convalescent patient plasma and monoclonal antibodies for therapy and/or prevention of severe disease and for comparing whether different vaccines generate antibodies that enhance monocyte infection and inflammation.

## Methods

### Human subjects

#### Fresh PBMCs and plasma cohort

The study was approved by the Investigation Review Boards of Boston Children’s Hospital and Massachusetts General Hospital (MGH), and all enrolled patients signed an informed consent. 35 patients 18 years or older with clinical symptoms suggestive of COVID-19 infection were enrolled at the time of presentation to the MGH emergency department (ED) from 7/9/20 to 01/12/21. A 10-ml EDTA blood sample was transported to Boston Children’s Hospital and processed within 2 h of collection. Only samples from patients with qRT-PCR verified SARS-CoV-2 infection were included in the study (31). Demographic and clinical data are summarized in Table S1. Healthy donor (HD) samples were processed and analyzed in parallel with patient samples.

#### Frozen plasma cohort

60 patients 18 yr or older with clinical symptoms suggestive of COVID-19 infection were enrolled in the MGH ED from 3/15/20 to 4/15/20 with an IRB-approved waiver of informed consent. Enrolled patients had at least one of the following: (i) tachypnea ≥22 breaths per minute, (ii) oxygen saturation ≤92% on room air, (iii) requirement for supplemental oxygen, or (iv) positive-pressure ventilation. A 10-ml EDTA tube was obtained with the initial clinical blood draw in the ED (n=60). Blood was also obtained on days 3 (n=42) and 7 (n=35) if the patient was hospitalized on those dates. Clinical course was followed for 28 d post-enrollment or until hospital discharge if after 28 d. SARS-CoV-2-confirmed patients (by qRT-PCR) were assigned a maximum acuity score (A1-A5) (A1 – died, A2 – required mechanical ventilation, A3 – hospitalized requiring supplemental oxygen, A4 – hospitalized but not requiring supplemental oxygen, A5 – discharged and not requiring hospitalization)^19,20^. Patients were grouped based on their worst acuity score over 28 d and divided into three groups for comparison (A1 and A2, severe disease; A3, moderate disease; A4 and A5, mild disease). Only 1 patient was in A4; therefore, most mild patients represent those that were discharged directly from the ED and thus have only a day 0 sample. Demographic and clinical data are summarized for each outcome group (Table S2).

#### Lung tissue specimens

Lung samples from 3 individuals who died from COVID-19 and 1 individual who died from trauma and without lung disease were obtained from Massachusetts General Hospital (MGH). The study was approved by the institutional review board of MGH IRB # 2020P001147. Informed consent was obtained from relatives of study participants. Lung tissue specimens were obtained within 24 h of autopsy and immediately formalin fixed and embedded in paraffin.

##### Plasma, PBMC and monocyte isolation

Samples were processed using recommended safety precautions in a BSL-2+ facility. Blood tubes were centrifuged at 2000 rpm for 10 min to separate plasma from blood cells. Plasma was collected to a new tube and incubated or not with 1% Triton X-100 for 1 h on ice before aliquoting and freezing at −80°C. Blood cells were resuspended in PBS and layered over Ficoll for density centrifugation. PBMC were collected from the interface and subjected to red blood cell lysis (if necessary) with Red Blood Cell Lysing Buffer Hybri-Max for 5 min on ice, followed by quenching with RPMI medium supplemented with 10% FBS and 1% Penicillin/Streptomycin. PBMC were washed once more with RPMI and one fraction was stained for flow cytometry, while the remaining cells were used for monocyte purification by magnetic separation using CD14^+^ magnetic beads. Sources of reagents are described in Table S3.

##### Multiplex Luminex, ELISA and LDH activity assay

IL-1β, IL-1RA, IL-2, IL-4, IL-5, IL-6, IL-7, IL-10, IL-12, IL-13, IL-17, IL-18, IL-21, IL-23, CCL3, CCL7, CCL9, CXCL10, G-CSF, TNF, IFN-β and IFN-γ were measured in plasma samples using a custom Luminex assay (R&D Systems), following the manufacturer’s instructions. Plates were analyzed using a Luminex MAGPIX Analyzer at the Analytical Instrumentation Core Lab of Boston University. GSDMD was measured in the same samples using the Human GSDMD ELISA kit (MyBiosource) following the manufacturer’s instructions, and LDH activity was measured using the CytoTox 96 Non-Radioactive Cytotoxicity Assay (Promega). Results from the latter assays were analyzed using a Biotek Synergy 2 analyzer; GSDMD absorbance was measured at 450 nm and LDH absorbance was measured at 490 nm.

##### Monocyte treatment and culture

Purified monocytes and THP-1 cells (ATCC) as controls, were cultured in RPMI + 10% FBS + 1% Pen/Strep for 30 min at 37°C in the presence or absence of 20 μM nigericin. Following incubation, monocytes were washed and used for FLICA assay, or fixed for 10 min with 4% PFA, washed twice with PBS + 3% FBS and kept at 4° C in PBS + 3% FBS until imaging flow cytometry or confocal microscopy.

As a positive control for pyrin activation, HD monocytes were cultured overnight with 2 μg/ml C3 Transferase from Clostridium botulinum (Rho I inhibitor), then fixed for 10 min with 4% PFA before staining for imaging flow cytometry (data not shown).

##### Intracellular staining for imaging flow cytometry and confocal microscopy

Fixed monocytes were permeabilized with 0.1% Triton X-100 for 10 min and washed twice with PBS + 3% FBS. Monocytes were then blocked for 30 min with PBS + 5% FBS, washed twice and then stained with unconjugated primary antibodies for ASC (1:200, mouse or rabbit), NLRP3 (1:200, goat), AIM2 (1:200, mouse), GSDMD (1:200, mouse), pyrin (1:200, rabbit), dsRNA (J2, mouse) (1:500) or SARS-CoV-2 nucleocapsid protein (1:500, rabbit) for 2 h, followed by 3 washes with PBS + 3% FBS. Cells were then stained with secondary antibodies (donkey anti-mouse, rabbit or goat conjugated with AlexaFluor 488, 546 or 647, at 1:1000) for 1 h in PBS + 3% FBS, followed by 3 washes.

For microscopy, cells were then stained with DAPI (1:1000) for 10 min, washed 3 times and cytospun onto glass slides (VWR); sealed using polyvinyl alcohol and 1.5 mm coverslips (VWR). Confocal images were acquired using a Zeiss LSM 800 with 405, 488, 561 and 633 nm lasers (emission filters, 465, 509, 561 and 668 nm, respectively) and a 63x 1.4NA oil immersion objective. Images were processed using Zen Blue 3.2.

For imaging flow cytometry, cells were resuspended in PBS + 3% FBS for analysis. Data were acquired using an ImageStream X MKII (Amnis) with 63x magnification and analyzed using Ideas software (Amnis). Monocytes were gated based on area/aspect ratio. ASC, NLRP3, AIM2 and pyrin specks were gated and quantified based on fluorophore intensity/max pixels.

##### Flow cytometry

PBMC were washed and stained for viability with Zombie Yellow in PBS (1:200) for 15 min on ice. Cells were washed with PBS, centrifuged, and then stained with Annexin V PE (1:200) in 1x Annexin Buffer for 15 min on ice. After washing with 1x Annexin V buffer, cells were blocked for 10 min with anti-CD32 (1:100) in PBS + 3% FBS, and then stained for 15 min on ice with a cocktail of antibodies to identify lymphocyte and myeloid cell subsets (all 1:200 except CD19 BV650, CD123 PerCP-Cy5.5 and CD56 APC-Cy7, 1:100). Purified monocytes and an A549 cell line overexpressing ACE2 were blocked with anti-CD32, then stained with primary antibodies for ACE2 (1:100) for 15 min on ice. The secondary anti-goat AF488 was coincubated with CD14 PE-Cy7 (1:200) and CD147 APC (1:100). After the last wash, cells were resuspended in 2% PFA and kept at 4°C until flow cytometry analysis. In vitro-infected monocytes were fixed and permeabilized with 0.1% Triton X-100, then blocked with PBS + 5% FBS. Cells were stained with primary antibodies for dsRNA (J2, mouse) (1:500), then stained with secondary antibody (donkey anti-mouse conjugated with AlexaFluor 647, at 1:500) and anti-CD14 PE-Cy7. Cells were acquired using a FACS Canto II or LSR II and data were analyzed using FlowJo Version 10.

##### FLICA assay

Monocytes, cultured or not for 30 min with nigericin, were washed and resuspended in RPMI 10% FBS with FLICA substrate (BioRad FAM-FLICA Caspase-1 kit), and cultured for 1 h at 37° C. Cells were then washed twice with 1X Apoptosis Buffer (from the kit) and fixed with 1x Fixative (from the kit). Cells were kept at 4°C until further staining and analysis.

##### Immunoblot

Lysates of enriched monocytes from HD and COVID-19 patients, the former treated or not for 16 h at 37°C with 100 ng/ml LPS and 20 μM nigericin, were resolved on 12% SDS PAGE gels, transferred to nitrocellulose membranes and blotted to detect GSDMD using (Abcam ab210070) primary rabbit mAb and secondary anti-rabbit IgG. Blotting for β-actin and COX-IV were used as loading controls.

##### eQTL analysis

To assess whether a causal association exists between *GSDMD* and other immune gene eQTLs and severe COVID-19, *in silico* analyses were performed using two sample Mendelian randomization ^65^ in R v4.0.2 ^66^ using the *TwoSampleMR* package ^67^. Mendelian randomization is a form of instrumental variable analysis that exploits the random allocation of alleles at meiosis to draw causal inferences using observational data by attempting to emulate randomization procedures that would be adopted in a clinical trial.

Uncorrelated single-nucleotide polymorphisms (SNPs) (r^2^<0.001 in European ancestry individuals in the 1000 Genomes Project, Phase 3 release) were associated with whole-blood RNA expression of *GSDMD* and other immune genes at genome-wide significance (P<5×10^−8^) from the eQTLGen consortium ^21^. These SNPs were cross-referenced against a large phenotypic database of publicly available genetic associations to ensure that they were not associated with potential confounding factors ^68,69^. Summary statistics from a genome-wide association study ^70^ of severe COVID-19 with respiratory failure were used for outcome data ^22^. Analysis was performed with data from release #4 (October, 2020) in which there were 4336 severe COVID-19 patients versus 623,902 control subjects, 6406 hospitalized COVID-19 patients versus 902,088 control subjects, and 269 severe COVID-19 patients vs 688 hospitalized COVID-19 patients. These analyses were based on different sample sets depending on whether the original investigators collected the relevant information either when planning the study or were able to obtain it retrospectively. In particular 14 studies contributed data to “COVID-19 hospitalized patients vs control population” and 12 studies contributed to “severe COVID-19 patients vs control population”. The control populations were a mix of subjects who were not COVID-19 infected (ie, negative test result(s)) or were assumed to be not COVID-19 infected (ie, there was no record of Covid-19 in their linked data).

CrossMap ^71^ was used to convert genomic positions from hg38 (as reported in the COVID-19 GWAS) to hg19 using the UCSC liftover chain file to ensure both the exposure and outcome datasets were reported on the same genome assembly. Variants were aligned so that the effect alleles were consistent across studies. The proportion of variance in expression of selected immune genes in whole blood explained by the selected SNPs the expected *F* value to examine potential weak instrument bias were then calculated ^72^.

Our primary analysis was based on the inverse variance weighted method of performing Mendelian randomization (this method combines the causal effect estimates from each individual genetic variant, computed as the ratio of the variant-expression association to the variant-Covid-19 association, into a single causal effect). A range of sensitivity analyses were performed relaxing some of the stricter assumptions underlying this method including the weighted median, modal and MR-Egger methods ^73^. If only a single genetic variant was selected for a gene, the Wald ratio method was used. The expected *F* value for *GSDMD* was 504.9 (with a lower limit of the one-sided 95% confidence interval (95% CI) of 462.1 indicating that considerable weak instrument bias would not be expected). There was no evidence of vertical pleiotropy (MR-Egger intercept p-value = 0.99) and findings were consistent across all sensitivity analyses.

Effect estimates are presented as odds ratios per standard deviation increase in *GSDMD* expression. A p-value < 0.05 was considered significant. All summary data used in this work are publicly available, together with a description of relevant participant consent and ethical approval secured in the original investigation.

##### Immunofluorescence (IF) of lung specimens

Formalin fixed and paraffin embedded lung parenchymal samples were stained for SARS-CoV-2 nucleocapsid (N), ASC, and CD14 and IF was analyzed on the Leica Bond RX automated staining platform using the Leica Biosystems Refine Detection Kit (Leica). The antibody for SARS Nucleocapsid (Novus) was run with citrate antigen retrieval and tagged with Alexa Fluor 488 Tyramide (Life). Following citrate stripping, the antibody for CD14 (Cell Signaling) was incubated and tagged with Alexa Fluor 594 Tyramide (Life). Following EDTA stripping, staining for ASC (Santa Cruz) was analyzed using antibody tagged with Alexa Fluor 647 Tyramide (Life). Samples were counterstained with DAPI. Slides were scanned using an Aperio Versa Digital Pathology Scanner (Leica) and analyzed with Aperio ImageScope v12.4.3 software (Leica). Slides were also analyzed by confocal microscopy as described above.

##### In vitro SARS-CoV-2 infection of HD monocytes

icSARS-CoV-2-mNG (a molecular clone of SARS-CoV-2 expressing Neon Green fluorescent protein) was a gift to AEG from Shi Pei Yong and the World Reference Center for Emerging Viruses and Arboviruses, Department of Microbiology and Immunology, University of Texas Medical Branch, Galveston, TX)^51^. The NG fusion protein is only expressed during viral replication. HD monocytes (purified from apheresis leukoreduction collars collected at Brigham and Women’s Hospital) were incubated overnight with medium or 100 ng/ml LPS, and then infected with icSARS-CoV-2-mNG (MOI =1) in a BSL-3 facility. The viral inoculum was treated with 10 μg/ml of antibody (isotype control mAb114, anti-Spike C1A-H12, or anti-Spike C1A-B12), or 10% HD or COVID-19 patient pooled plasma (heat inactivated or not; Ig-depleted or not, as indicated) before infection with SARS-CoV-2 for 30 min at room temperature. 100 μl of treated virus was added to monocytes (2x10^6^ cells/well) in 48 well plates. Infected cells were incubated at 37°C, 5% CO_2_ with gentle shaking every 10 min for 1 h, after which the culture volume was increased to 500 μl with RPMI supplemented with 5% heat inactivated normal AB human serum and 10 μg/ml of the aforementioned antibodies or 10% pooled HD or COVID-19 patient plasma. Cultures were then incubated at 37°C, 5% CO_2_ for 48 h at which time cells were harvested and fixed for 20 min with 4% PFA and then stained. Immunoglobulin (Ig) from COVID-19 patient pooled plasma was depleted by protein A/G agarose resin. Control samples were incubated with agarose resin without coupled protein. C1A-B12 and C1A-H12, two SARS-CoV-2 Spike-targeting human monoclonal antibodies, were produced as previously described ^52^.

##### qRT-PCR

RNA was extracted using Trizol reagent (Invitrogen) from COVID patient monocytes or from uninfected or infected HD monocytes (stimulated or not with LPS (100 ng/ml for 16 h)), then reverse transcribed using a High Capacity cDNA Reverse Transcription Kit (Applied Biosystems). Random primers were used to generate cDNA for detection of cellular RNAs (*ACE2, BSG, ACTB*) and SARS-CoV-2 specific primers were used to generate cDNA to detect viral genomic RNAs (N1 region of *N* gene). cDNA was analyzed by qRT-PCR using the Sso Fast EvaGreen Supermix (BioRad) (30 sec at 95°C, 40 cycles (3 sec at 95°C; 3 sec at 54 °C) using a CFX96 Touch Real-Time PCR Detection System (BioRad). To detect SARS-CoV-2 subgenomic RNA, qRT-PCR was carried out using a primer pair with the forward primer annealing to the 5’ leader region of the viral genome and the reverse primer annealing to the 3’ UTR. With the cycling conditions used (30 sec at 95 °C, 40 cycles (30 sec at 95°C, 30 sec at 60 °C, 90 sec at 72 °C)), full-length genomic RNA was not amplified, but small subgenomic RNA segments (<3 kB) could be amplified ^30,74^ For each sample, Ct values were normalized to the *ACTB* Ct value. Primer sequences are given in Table S4. Subgenomic RNA qPCR products were also analyzed by electrophoresis on 1% agarose gels stained with ethidium bromide and visualized on a Chemidoc imager (BioRad). The ~1600 nt band was excised and sequenced to confirm its origin as the SARS-CoV-2 subgenomic RNA encoding for N.

##### Plaque assays

Vero E6 cells were seeded as monolayers in 24-well plates 1 d prior to infection. Virus-infected sample culture supernatants were serially diluted in DMEM. The plates were washed once with DPBS and then infected with 100 μl of diluted sample and incubated at 37 °C, 5% CO_2_ for 1 h with rocking every 15 min. After 1 h, the inoculum was removed and an overlay of 1% methylcellulose (Sigma) in complete MEM (Gibco) was applied to each well. The plates were incubated at 37 °C until plaques were observable in positive control wells. To visualize plaques, the overlay was removed and the cell monolayer was fixed with 4% PFA and stained with crystal violet. Plaques were then counted to quantify the virus titer in PFU/ml.

##### Anti-Spike RBD ELISA

Enzyme-linked Immunosorbent Assay (ELISA) kit anti-Spike RBD (BioLegend) was used to quantify antigen-specific IgG in plasma from HD and COVID-19 patients. ELISA was performed as per manufacturer’s instructions. Anti-Spike RBD absorbance was measured at 450 nm and quantified by linear regression based on the standard curve.

##### Statistical Analysis

Statistical analysis was performed using GraphPad Prism V7.0. Normal distribution of the data was evaluated by the D’Agostino and Pearson normality test prior to applying statistical methods. Distributions were considered normal if *P* ≤ 0.05. Parametric or non-parametric (Mann-Whitney or Kolmogorov-Smirnov tests) two-tailed unpaired *t*-tests were used to compare two unpaired groups. Multiple group comparisons were analyzed by one-way ANOVA with Sidak’s or Tukey’s multiple comparisons tests, or non-parametric Kruskal-Wallis with Dunn’s post-test. Multiple groups were compared by two-way ANOVA with additional Sidak’s or Tukey’s multiple comparisons test. Mean plasma values from hospitalized COVID-19 patients on each day were compared between severity groups by multiple unpaired *t*-tests. Correlations of plasma levels were determined by simple linear regression and Pearson correlation coefficient.

## Extended Data

## Supplementary Material

Supplement 1 **Supplemental Table S1: Demographic and clinical information of the fresh PBMCs and plasma cohort**. Age, race and ethnicity, body mass index, co-morbidities, symptoms, MGH Acuity score, hospitalization details and clinical information of the patients in the fresh PBMCs and plasma cohort.

Supplement 2 **Supplemental Table S2: Demographic and clinical information of the frozen plasma cohort.** Age, body mass index, co-morbidities, symptoms, MGH Acuity score, hospitalization details and clinical information of the patients in the frozen plasma cohort.

Supplement 3 Supplemental Table S3: eQTL data

Supplement 4 **Supplemental Table S4: Reagents and materials used for this manuscript.** Antibodies, chemicals and commercial kits (with sources and catalog numbers) described in Methods.

## Data Availability

The data and materials that support the findings of this study are available from the corresponding authors upon request.
